# Frontier-based techniques in measuring hospital efficiency in Iran: a systematic review and meta-regression analysis

**DOI:** 10.1186/1472-6963-13-312

**Published:** 2013-08-15

**Authors:** Aliasghar A Kiadaliri, Mehdi Jafari, Ulf-G Gerdtham

**Affiliations:** 1 Division of Health Economics, Department of Clinical Sciences-Malmö, Lund University, Malmö University Hospital, CRC, Entrance 72, House 28, Plan 10, Room 027, 20502 Malmö, Sweden; 2 Department of Health Management and Economics, School of Public Health, Tehran University of Medical Sciences, Tehran, Iran; 3 Health Economics & Management, Institute of Economic Research, Lund University, Lund, Sweden; 4 Department of Health Management and Economics, School of Health Informatics and Management, Iran University of Medical Sciences, Tehran, Iran; 5 Department of Economics, Lund University, Lund, Sweden

**Keywords:** Technical efficiency, Meta-regression, Data envelopment analysis, Stochastic frontier analysis, Iran

## Abstract

**Background:**

In recent years, there has been growing interest in measuring the efficiency of hospitals in Iran and several studies have been conducted on the topic. The main objective of this paper was to review studies in the field of hospital efficiency and examine the estimated technical efficiency (TE) of Iranian hospitals.

**Methods:**

Persian and English databases were searched for studies related to measuring hospital efficiency in Iran. Ordinary least squares (OLS) regression models were applied for statistical analysis. The PRISMA guidelines were followed in the search process.

**Results:**

A total of 43 efficiency scores from 29 studies were retrieved and used to approach the research question. Data envelopment analysis was the principal frontier efficiency method in the estimation of efficiency scores. The pooled estimate of mean TE was 0.846 (±0.134). There was a considerable variation in the efficiency scores between the different studies performed in Iran. There were no differences in efficiency scores between data envelopment analysis (DEA) and stochastic frontier analysis (SFA) techniques. The reviewed studies are generally similar and suffer from similar methodological deficiencies, such as no adjustment for case mix and quality of care differences. The results of OLS regression revealed that studies that included more variables and more heterogeneous hospitals generally reported higher TE. Larger sample size was associated with reporting lower TE.

**Conclusions:**

The features of frontier-based techniques had a profound impact on the efficiency scores among Iranian hospital studies. These studies suffer from major methodological deficiencies and were of sub-optimal quality, limiting their validity and reliability. It is suggested that improving data collection and processing in Iranian hospital databases may have a substantial impact on promoting the quality of research in this field.

## Background

According to the World Health Organization (WHO), the percentage of health expenditure out of gross domestic production (GDP) in Iran increased from 4.6% in 2000 to 5.5% in 2008 [1]. As hospitals are considered the main consumer of health care resources in any health care system [2], the issue of containing costs without loss of quality has been high on the health care agenda in most countries. In this regard, improving efficiency is considered a main option in planning to contain hospital costs [3].

In 2006, bed occupancy rate and average length of stay for the hospitals affiliated to the Iranian Ministry of Health were 70% and 3.38 days, respectively [4]. In comparison with international figures, these figures indicate technical inefficiency in hospitals in Iran and there is much scope for improvement. This inefficiency has attracted the attention of policy-makers, resulting in measures such as formation of a Board of Trustees in hospitals, implementation of performance-based budgeting, establishing a hospital information system and maintenance management [5]. In response to this policy interest, a considerable body of literature has emerged to measure the efficiency of Iranian hospitals in recent years. As these studies are mainly policy-oriented, and aim to assist policy-making, the estimated efficiency scores should be robust to model specifications [6] or policy-makers should at least be aware of the effects of these specifications on estimated scores.

Efficiency can be assessed in term of different concepts including technical, scale, price and allocative efficiency [7]. Efficiency concept used in our review is technical efficiency which is a measure based on work of Farrell [8]. A hospital is technically efficient when it is producing the maximum amount of output from a given amount of input, or alternatively producing a given output with minimum quantities of inputs. Thus, when a hospital is technically efficient, it operates on its production frontier [9].

Different methods have been applied to measure hospital efficiency around the world, the frontier-based methods, mainly data envelopment analysis (DEA) and stochastic frontier analysis (SFA), being the most common [10]. The frontier-based methods compare hospitals’ actual performance against an estimated efficient frontier. It is well documented that the features of frontier-based methods have an important impact on the estimated efficiency scores [6,10-16]. Selection of input and output variables is among the main features which significantly affect the results of these models [17,18]. In addition, the selection of input and output variables in these studies may negatively affect individual and population health (e.g. using length of stay as output may encourage hospitals to admit patients with less complicated disease [18]).

On the output side, there are two main types of outputs: health services (e.g. number of outpatient visits, number of inpatient visits, etc.) and health outcomes (e.g. post-operative mortality rate, blood pressure control, etc.) [10]. In practice, due to lack of data, most studies use health services as a proxy for health outcomes [10]. These studies implicitly assume that health services lead to health outcomes and no difference is measured between hospitals in providing health services [6]. However, if this assumption does not hold, then using health services as a proxy for health outcomes is problematic [15]. For example, if a hospital provides health services of low quality which lead to adverse events and re-admission, then using inpatient days as output would mean rewarding this hospital for its bad performance. Moreover, this approach generally ignores hospitals’ functions other than curative care (e.g. prevention, research and educational functions) [18]. In addition, many earlier studies in developed countries and most studies in developing countries naively used raw counts in capturing the health services as output. This can lead to bias if there are case-mix differences between hospitals [6,18].

Regarding input, there are three main input categories: human (e.g. physicians, nurses), capital (e.g. beds), and consumable resources (e.g. consumed drugs). These variables are generally measured as counts (number of physicians, nursing hours) or as monetary values (e.g. salaries, annual expenditure of capital) [6]. In addition, features such as sample size, homogeneity of units under study, ratio between sample size and number of input and output variables, and input/output returns-to-scale orientation also affect the estimated efficiency scores [6,10-16].

Although there are several systematic reviews of health care efficiency studies in the literature [10,17-22], to our knowledge only one previous study [6] has used meta-regression to quantify the impact of modelling choices on the estimated efficiencies in reviewed studies. As all of these studies focused on English-language publications, many of non-English studies were overlooked in these systematic reviews. Moreover, some recent Iranian studies published in English were not included in these systematic reviews. Hence, the contribution of the current study is to systematically review hospital efficiency studies in Iran, published in Persian and English, and quantify the impact of modelling choices on the estimated efficiency scores using meta-regression.

## Methods

### Search strategy

A literature review by AAK and MJ was independently conducted by searching international (EconLit, Pubmed, Scopus, Embase, and Web of Science) and national Iranian (SID, Magiran) databases in September 2011 with an update performed in January and November 2012. Search terms included efficiency, hospital, productivity, DEA, SFA, and Iran. The PRISMA guidelines [23] were followed in the search process.

### Selection of studies

Five inclusion criteria were applied: (1) the report included mean technical efficiency (TE) or data needed to calculate it; (2) the unit of analysis was the hospital; (3) the data required for analysis were available (by access to the full text of the publication or by request from the author); (4) the study’s observations were limited to hospitals within the boundaries of Iran; (5) the report was published in Persian or English.

**Figure 1:**
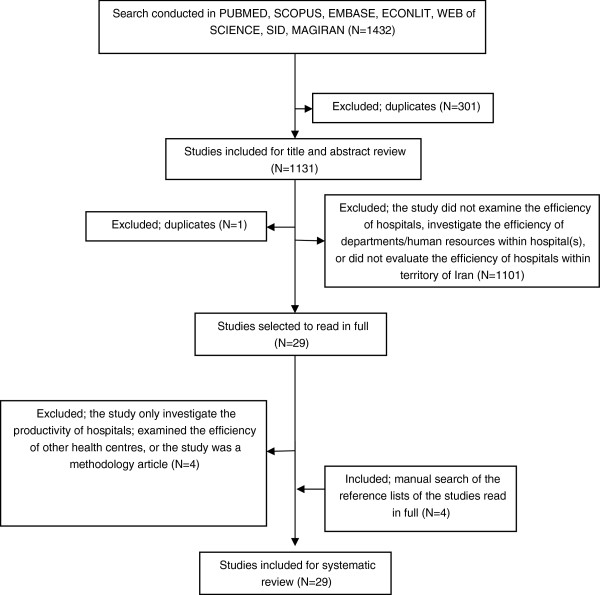
Flow diagram of literature search.

The initial search resulted in 1,432 documents. After excluding duplicates and non-relevant studies, 29 articles were selected for full text examination. The reference lists of these 29 documents were manually searched. In total, 29 studies passed the exclusion criteria for the systematic review. In addition to peer-reviewed articles we also included MSc and PhD dissertations and theses and conference proceedings in the analysis. Figure 1 shows the process of study selection.

### Data extraction

For each study, data on the year and language of publication, number of hospitals included in the study, estimation methods, activity of the hospitals (teaching and/or non-teaching), ownership status of the hospitals (government, private, social security organization, charity and military), type of hospital (general and/or specialty), data years, geographical coverage of the study (single or multiple provinces), number of variables (inputs and outputs) used in the model and estimated efficiency scores were extracted.

### Statistical analysis

Two types of analyses were applied: univariate and multivariate. In the univariate analysis, the mean TE was compared using Wilcoxon’s rank-sum test based on different features of the studies. In the multivariate analysis, the mean TE was used as dependent variable in the meta-regression. Based on literature and model specifications in the primary studies, we used the number of variables (dimension), sample size (number of hospitals), estimation method (SFA v. DEA), orientation (input v. output), percentage of teaching hospitals, publication period and heterogeneity in the sample in terms of type, activity, location and ownership as explanatory variables. We included these variables because previous studies showed that heterogeneity across the sample can affect the estimated efficiency scores [15]. Moreover, as all the studies which used panel data reported a separate score for each year, we averaged these estimates and calculated a pooled TE and included a dummy variable in our meta-regression as pooled v. single estimate.

The linear-log function, as recommended by Nguyen and Coelli [6], was used in the following estimation:

$$\begin{array}{l} \mathrm{MTE}=\beta_{0}+\beta_{1}\left(\mathrm{SFA}\right)+\beta_{2}\left(\text{Pooled}\right)+\beta_{3}\ln\left(\text{Size}\right)\\ \;+\beta_{4}\ln\left(\text{Dimension}\right)+\beta_{5}\left(\text{Output}-\text{orient}\right)\\ \;+\beta_{6}\left(\text{Heterogeneity}\right)+\beta_{7}\left(\text{Publication}\;\text{period}\right)\\ \;+\beta_{8}\left(\%\;\text{of}\;\text{teaching}\;\text{hospitals}\right) \end{array}$$

where MTE is the mean TE. For the estimation method, the ordinary least square (OLS) method was used as it is a consistent estimator and is preferred to the Tobit model in efficiency analyses [24,25]. Moreover, as there were no efficiency scores equal to 1 in our dataset, the Tobit regression produces the same estimates as OLS regression [26]. The standard errors were corrected for clustering when estimates were derived from the same study and also for heteroskedasticity [27]. Data were analysed using STATA statistical package, version 11 (StataCorp LP, College Station, TX, USA).

### Sensitivity analysis

To assess the robustness of the results, a sensitivity analysis was conducted excluding studies that were not journal articles. Moreover, to check the influence of each study on the results, a sensitivity analysis was performed omitting each study in turn and then estimating the summary effect of the remaining studies.

## Results

**Figure 2:**
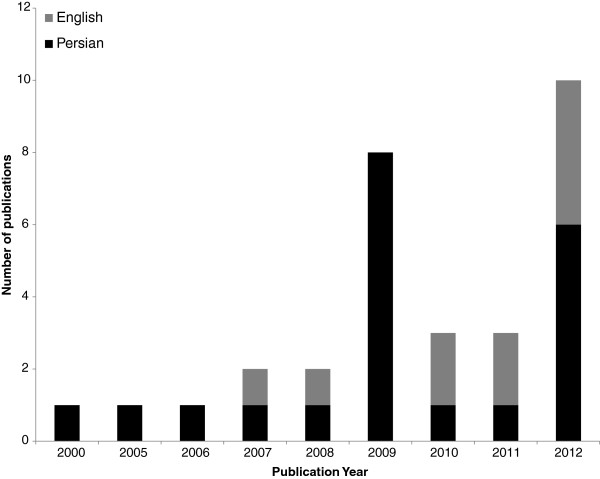
Distribution of studies by year and language.

A total of 43 estimated efficiency scores from 29 studies were retrieved and included in the meta-regression analysis. Table 1 shows the characteristics of studies included in our analysis. There was a 5-year lag between the first and second applications of frontier-based methods in measuring hospital efficiency in Iran. After the second study using this method, in 2005, there was at least one publication per year on the topic, with a peak in publications in 2012 (Figure 2). The years of data used in the estimation ranged from 1996 to 2010. Sample size ranged from four to 122, with a median of 16 hospitals per model. The dimension (inputs and outputs) ranged from four to ten, with a median of six variables per model. Data envelopment analysis was applied in all studies, with three studies using both DEA and SFA to estimate the efficiency scores. Most studies (25 out of 29) were carried out in a single province of Iran. Among the provinces, the hospitals in Tehran (Iran’s capital) were naturally studied more than the other hospitals. DEAP version 2.1 [28] and FRONTIER version 4.1 [29] were the main software packages used to estimate DEA and SFA models, respectively. All studies were input-oriented and two studies used output orientation in a sensitivity analysis.

**Table 1:** The characteristics of studies included in the meta-regression analysis

**No.**	**Author(s)**	**Publication year**	**Publication language**	**Data year**	**No. of hospitals**	**Proportion of teaching hospitals (%)**	**Number of input/output variables**	**Estimation method**	**Province**	**Software used to assess efficiency**	**Number of estimates**
1	Abolhalaj et al. [30]	2011	Persian	2007	122	45.08	4/2	DEA	NA	DEAP 2.1	6
2	Ahmadkiadaliri et al. [31]	2011	English	2006	19	21.05	2/4	DEA	Khuzestan	DEAP 2.1	1
3	Akbari et al. [32]	2012	Persian	2005–2008	20	NA	4/3	DEA	East Azerbaijan	DEAP 2.1	1
4	Alemtabriz & Imanipour [33]	2009	Persian	2005–2007	16	68.75	4/5	DEA	Tehran	LINDO	1
5	Ardakani et al. [34]	2009	Persian	2004–2006	12	33.33	3/3	DEA	Yazd	LINDO	1
6	Asadi et al. [35]	2012	Persian	2008	13	38.46	3/3	DEA	Yazd	DEAP 2.1	1
7	Askari et al. [36]	2012	Persian	2001–2009	13	38.46	4/3	DEA	Yazd	DEAP 2.1	1
8	Farzianpour et al. [37]	2012	English	2008–2010	16	100.00	3/3	DEA	Tehran	GAMS	1
9	Goudarzi [38], Ghaderi et al. [39]*	2006, 2007	Persian	2000–2004	26	42.31	4/4, 4/1	DEA, SFA	Tehran	DEAP 2.1, Frontier 4.1	2
10	Goudarzi et al. [40]	2012	Persian	2001–2007	13	30.77	4/5, 4/1	DEA, SFA	Lorestan	DEAP 2.1, Frontier 4.1	2
11	Hajialiafzali et al. [41]	2007	English	2002	53	0.00	4/4	DEA	NA	DEASOFT-V1	1
12	Hatam [42]	2008	English	1996	18	0.00	2/4	DEA	NA	NA	1
13	Hatam et al. [43]	2010	English	2005–2006	21	NA	3/5	DEA	Fars	GAMS	1
14	Pourmohammadi [44] Hatam et al. [45]*	2009, 2012	Persian & English	2006–2008	64	0.00	4/4, 4/1	DEA, SFA	NA	WIN4DEAP, Frontier 4.1	2
15	Ilbeigi et al. [46]	2012	Persian	2009	17	NA	2/3	DEA	Khorasan Razavi	DEAP 2.1	1
16	Jandaghi et al. [47]	2010	English	2008	8	NA	2/3, 3/3, 5/3, 4/3	DEA	Qom	WIN4DEAP	4
17	Marnani et al. [48]	2012	English	2010	23	56.52	1/4	DEA	Tehran	WIN4DEAP	1
18	Pourreza et al. [49]	2009	Persian	1996–2006	12	83.33	4/4	DEA	Tehran	DEAP 2.1	1
19	Rahimi et al. [50]	2012	Persian	2009	23	17.39	3/2	DEA	West Azerbaijan	DEAP 2.1	1
20	Rezapoor & Asefzadeh [51]	2009	Persian	1998–2007	4	100.00	2/5	DEA	Qazvin	DEAP 2.1	1
21	Sabermahani et al. [52]	2009	Persian	2007	13	23.08	4/3	DEA	Kerman	DEAP 2.1	1
22	Sajadi et al. [53]	2009	Persian	2005–2006	23	13.04	5/5	DEA	Isfahan	DEAP 2.1	2
23	Salehzadeh & Ketabi [54]	2011	Persian	2007	8	37.50	2/2	DEA	Qom	WIN4DEAP, DEA-Master	1
24	Shahhoseini et al. [55]	2011	English	2008	12	33.33	4/5	DEA	NA	DEAP 2.1	1
25	Sheikhzadeh et al. [56]	2012	English	2005	11	45.45	4/3	DEA	East Azerbaijan	DEAP 2.1	1
26	Ahmad Kiadaliri [57]*	2005	Persian	1996–2003	8	62.50	4/4, 4/1	DEA	Tehran	DEAP 2.1	2
27	Najafi [58]*	2008	Persian	2000–2006	12	33.33	2/2	DEA	Ardebil, Tehran	DEAP 2.1	1
28	Kazemi et al. [59]*	2009	Persian	2006–2008	11	18.18	2/2	DEA	Southern Khorasan, Northern Khorasan, Khorasan Razavi	DEAP 2.1	2
29	Zarei [60]*	2000	Persian	1999	57	68.42	6/4	DEA	Tehran	NA	1

*These are grey literature including thesis and conference presentations. Goudarzi and Pourmohammadi published their results of DEA application as a journal article [39,45].

In terms of ownership status of the hospitals, 20 out of 29 studies exclusively estimated the efficiency of government hospitals. Among the remaining studies, government hospitals were included in six studies. Regarding the hospitals’ activity, 23 studies included both teaching and non-teaching hospitals in the sample, while five studies included only non-teaching hospitals and one included only teaching hospitals. In terms of types of hospital, in 17 studies the sample included both general and specialty hospitals while twelve studies included only general hospitals.

Regarding input and output variables included in the studies, while human and capital resources were included in almost all studies (one study included the number of staffed beds as a single input), only four studies included consumable resources as an input variable. In most studies (93.1%), the input variables were measured as counts. In six studies, human resources were aggregated into one category. The number of staffed beds was used as the main proxy for capital resources. Generally a combination of number of physicians, number of nurses, number of other human resources and number of staffed beds were selected as the input variables.

Almost all studies (96.55%) considered only health services as the output variable. In three studies, annual hospital income was also included as an output variable. These variables were typically included as raw counts, such as inpatient days and number of surgeries, without adjusting to the differences in the severity of treated cases. Teaching and research activities of hospitals were not accounted for in most of the studies (only one study captured these).

Table 2 gives a quality assessment of the studies included in the analysis and shows aspects which might bias the estimated efficiency scores. Most of these aspects (seven out of nine), especially adjustment for quality of care and capturing hospitals’ teaching activities, were weakly handled in most of the studies. Only in about 40% of the studies was the rule of thumb of three observations per variable [61] satisfied. In seven studies, univariate statistical analysis (*t*-test) was applied to assess the relationship between efficiency scores and some environmental factors (Table 2, column 4). The sensitivity analysis through specifying models with different mix of variables to test the robustness of results was applied in only four studies.

**Table 2:** Quality assessment of studies included in the meta-regression analysis

**No.**	**Adjustment for quality of care?**	**Adjustment for teaching activities?**	**Assess the determinants of efficiency?**	**Sensitivity analysis?**	**Ratio of size to dimension ≥3?**	**Homogeneity in type of hospital (general/specialty)?**	**Homogeneity in activity (teaching/non-teaching)?**	**Homogeneity in hospital ownership status?**	**Homogeneity in location (same/multiple province)?**
1	No	No	No	No	Yes (in 5 out of 6 estimates)	Yes (in 5 out of 6 estimates)	Yes (in 5 out of 6 estimates)	Yes	No
2	No	No	No	No	Yes	Yes	No	Yes	Yes
3	No	No	Yes	No	No	Yes	No	Yes	Yes
4	No	No	No	No	No	Yes	No	Yes	Yes
5	No	No	No	No	No	No	No	Yes	Yes
6	Yes	Yes	No	No	No	No	No	Yes	Yes
7	No	No	Yes	No	No	No	No	Yes	Yes
8	No	No	No	No	No	No	Yes	Yes	Yes
9	No	No	Yes	No	Yes	No	No	Yes	Yes
10	No	No	Yes	No	No	No	No	Yes	Yes
11	No	–	No	No	Yes	No	Yes	Yes	No
12	No	–	No	No	Yes	No	Yes	Yes	No
13	No	No	No	No	No	Yes	No	Yes	Yes
14	No	–	No	No	Yes	No	Yes	Yes	No
15	No	No	No	No	Yes	Yes	No	Yes	Yes
16	No	No	No	Yes	No	No	No	No	Yes
17	No	No	No	No	Yes	No	No	Yes	Yes
18	No	No	Yes	No	No	No	No	Yes	Yes
19	No	No	No	No	Yes	No	No	Yes	Yes
20	No	No	No	No	No	Yes	No	Yes	Yes
21	No	No	Yes	No	No	Yes	No	Yes	Yes
22	No	No	No	Yes	No	Yes	No	Yes	Yes
23	No	No	No	No	No	No	No	No	Yes
24	No	No	No	No	No	No	Yes	No	Yes
25	No	No	No	No	No	No	No	No	Yes
26	No	No	No	Yes	No	Yes	No	Yes	Yes
27	No	No	No	No	Yes	Yes	No	Yes	No
28	No	No	Yes	Yes	No	No	No	No	No
29	No	No	No	No	Yes	No	No	No	Yes

**Figure 3:**
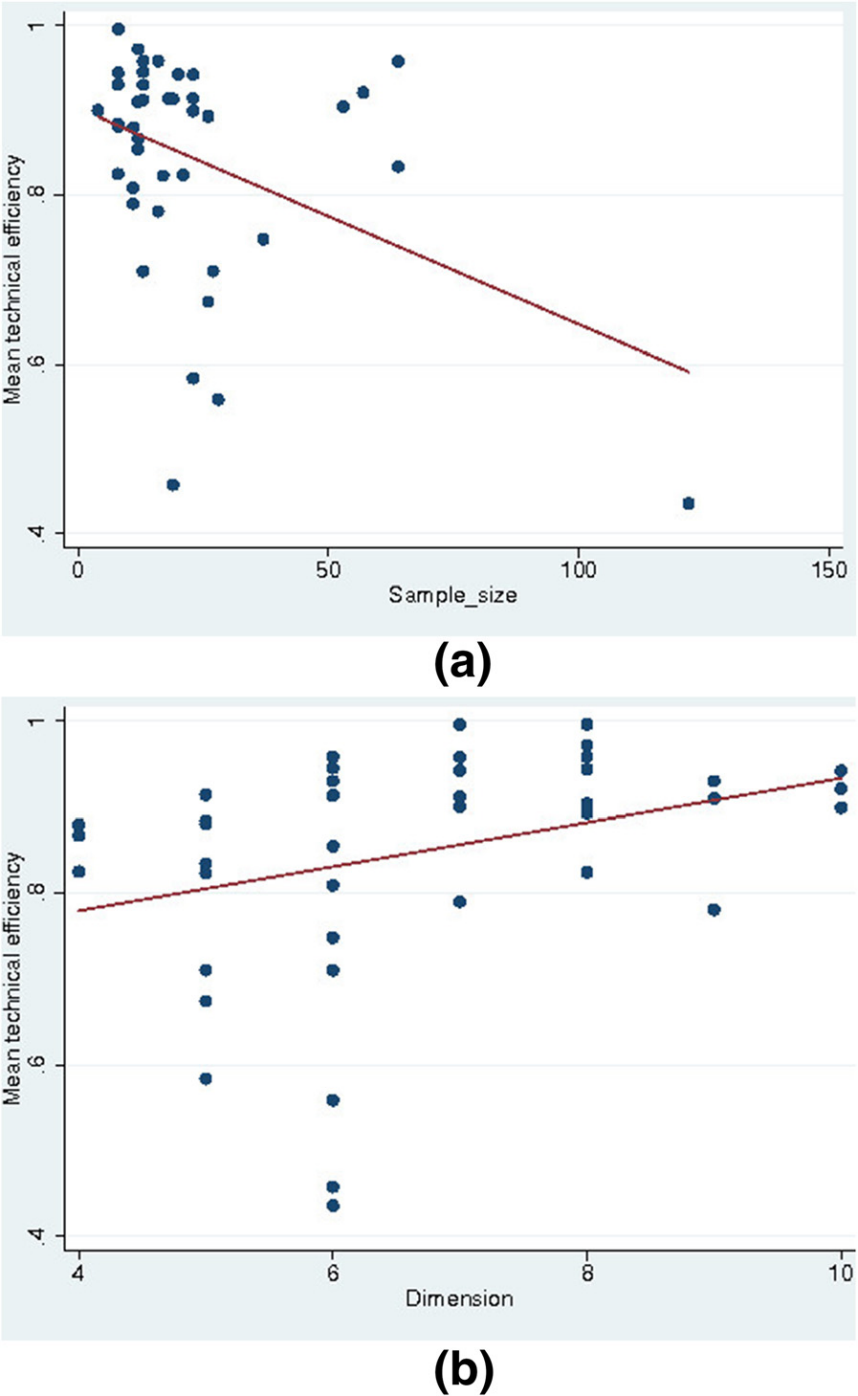
The relation between the mean technical efficiency and (a) size and (b) dimension.

Table 3 shows the mean TE according to different characteristics of the studies. The pooled estimate of mean TE was 0.846 (±0.134). This suggests that hospitals could improve their performance by about 15%. The maximum and minimum of efficiency scores were 0.436 and 0.996, respectively (not shown). This indicates a considerable variation in the efficiency scores between the different studies performed in Iran. The studies that used SFA for estimation reported lower efficiency scores compared with studies using DEA, but this difference was not statistically significant. Studies that included hospitals from a single province reported higher efficiency scores compared with cross-province studies. Larger sample size and lower number of input and output variables in the models were associated with lower efficiency scores. Figures 3a–b present the relation between mean TE and the number of variables (dimension) and sample size for each model.

**Table 3:** Mean technical efficiency (TE) by the variables used in the analysis

**Variable**	**No. of estimates**	**Mean TE (± SD)**	**p-value**
Method			
SFA	3	0.739 (±0.084)	0.060
DEA	40	0.854 (±0.134)	
Orientation			
Input	41	0.843 (±0.137)	0.885
Output	2	0.890 (±0.013)	
Estimation			
Pooled	20	0.872 (±0.083)	0.679
Single	23	0.822 (±0.164)	
Heterogeneity in location			
Yes	13	0.766 (±0.176)	0.015
No	30	0.880 (±0.095)	
Heterogeneity in activity			
Yes	32	0.863 (±0.120)	0.290
No	11	0.797 (±0.165)	
Heterogeneity in ownership			
Yes	10	0.901 (±0.066)	0.307
No	33	0.829 (±0.145)	
Heterogeneity in type of hospital			
Yes	26	0.863 (±0.131)	0.109
No	17	0.819 (±0.137)	
No. of hospitals^a^			
≤16	23	0.892 (±0.073)	0.070
>16	20	0.793 (±0.167)	
No. of input and output variables			
≤6	24	0.791 (±0.151)	0.001
>6	19	0.914 (±0.060)	
No. of hospitals:No. of variables ratio			
<3	26	0.894 (±0.071)	0.017
≥3	17	0.771 (±0.172)	
Publication year			
≤2009	19	0.885 (±0.068)	0.406
>2009	24	0.815 (±0.164)	
Type of publication			
Journal article	35	0.842 (±0.144)	0.662
Other	8	0.861 (±0.082)	
Overall	43	0.846 (±0.134)	–

^a^ Divided based on median value.

Table 4 presents the results of the meta-regression analysis. Different models were applied and based on F-test results and R-square, model 7 was selected as the final model. Everything else equal, larger sample size was associated with lower efficiency scores. Evaluating the marginal effect of sample size in the median sample size of 16 yielded a marginal effect of −0.006. On the other hand, there was a positive association between dimension and the efficiency scores. The effect of dimension on estimated efficiency scores was more substantial than that of size. In the sample median of six variables, the marginal effect of dimension was equal to 0.03. While heterogeneity was associated with higher efficiency scores, only heterogeneity in type of hospital was statistically significant. Studies that were published from 2010 onwards reported, on average, 0.07 lower efficiency score compared with studies published before this year.

**Table 4:** Results of the meta-regression analysis

	**Model 1**	**Model 2**	**Model 3**	**Model 4**	**Model 5**	**Model 6**	**Model 7**	**Model 8**
Ln (Size)	−0.088*	−0.081	−0.077**	−0.083**	−0.080*	−0.094**	−0.097**	−0.101***
Ln (Dimension)	0.197***	0.174***	0.172**	0.195***	0.200***	0.216***	0.198***	0.204***
SFA		−0.065						
Pooled		0.060						
Output orientation		0.035						
Heterogeneity in location			−0.033					
Heterogeneity in activity				0.020				
Heterogeneity in ownership status					0.041			
Heterogeneity in type of hospital						0.069*	0.072**	0.069*
Published from 2010 onwards							−0.071**	−0.076**
Percentage of teaching hospitals in sample								−0.077
Constant	0.731 ***	0.667 ***	0.754***	0.705***	0.691***	0.669***	0.750***	0.779***
N	43	43	43	43	43	43	43	36
R-squared	0.290	0.336	0.298	0.294	0.305	0.354	0.423	0.456

SFA = stochastic frontier analysis.

***, ** and * shows the 1%, 5% and 10% significance level, respectively; standard errors adjusted for clustered observations.

When we included only the estimations from the journal articles in the sensitivity analysis, publication before 2010 was no longer significant and heterogeneity in type of hospital was significant at the 10% level. Moreover, another sensitivity analysis showed that no single study had a significant effect on our results.

## Discussion

In this analysis, we reviewed studies that measured TE of hospitals in Iran and quantified the impact of model specifications on the reported efficiency scores using meta-regression analysis based on 43 extracted observations from 29 different studies. To our knowledge, this is the first attempt to quantify the effect of model choice on hospitals’ efficiency scores in a developing country such as Iran. There has been a growing trend in recent years to measure hospitals’ efficiency using frontier-based techniques, especially DEA. The findings from the review study also show that many studies suffer from major methodological problems and are of sub-optimal quality.

We found that DEA was the dominant method of measurement of hospital efficiency in Iran. This is in line with previous international findings [20,21]. Ability to handle multiple inputs and outputs in different units of measurement is the main explanation for this [20]. In addition, similar to previous reviews [6,19], input orientation was the main choice in these studies, suggesting that hospital managers have more control over inputs than over outputs.

Aggregation of input categories, focus on curative function of hospitals, no adjustment for differences in case mix and quality of care between hospitals, small sample size, little adjustment for heterogeneity in the sample, and no attempt to examine the causes of inefficiency, as well as no attempt to evaluate the misspecification in applied models, and little attempt to analyse the relationship between the efficiency scores and environmental factors are some of the main, and common, limitations of these studies. This raises many issues of validity, usefulness and generalizability of these studies in Iran.

It seems that the lack of data is the main reason for these limitations among Iranian studies. As has been argued by Afzali et al. [18], Iranian hospital databases suffer from data limitation regarding a broad range of hospital functions (e.g. preventive care, health promotion) and quality of care. Hence, improving data collection and processing in Iranian hospital databases is a critical step in promoting quality in hospital efficiency studies. On the other hand, a few studies have tried to deal with these limitations, for example using the ratio of the number of major surgeries to the total number of surgeries to capture the complexity of surgical operations [41], or the ratio of published scientific articles to the total number of physicians to capture the hospitals’ research function [35]. This implies that available data are not always used appropriately by researchers in Iran, possibly due to a limited understanding of hospitals’ functions among researchers.

The results of the meta-regression show that there were no significant differences in the estimated efficiency scores between SFA and DEA applications. There is no agreement on the impact of parametric v. non-parametric method on the efficiency scores in the literature. Kontodimopoulos et al. [12] reported lower efficiency scores for DEA compared with SFA, while Gannon [62] found the opposite. On the other hand, Nguyen and Coelli [6] did not find any statistically significant differences between these two methods.

The associations between sample size and dimension and the estimated efficiency scores are in line with previous studies [6,16]. It is argued that small sample size may cause sparsity problems, meaning that a hospital can be deemed efficient just because of lack of any comparator. In other words, as sample size increases, the estimated efficient frontier asymptotically approaches the true frontier and more observations are deemed inefficient compared with a smaller sample size [16]. In the same way, everything else equal, increasing the number of input and output variables in a model raises the sparsity problem [16]. The estimated marginal effect of sample size and dimension in our study may help policy-makers to compare the results of different studies with different sample sizes and dimensions in the country. Including more heterogeneous hospitals in the sample is associated with higher efficiency scores, as previously confirmed by Jacobs et al. [15].

The findings of the current study should, however, be interpreted with caution. As Iranian databases are not well developed, we may have missed some studies. Because only a few studies used SFA, it was not possible to control for the model choices in SFA, such as functional form used for technology structure, distribution of inefficiency components, etc. There are some other explanatory variables (e.g. location of hospital, degree of autonomy) that may affect the estimated efficiency scores of different studies, but the small sample size did not allow us to control for them.

## Conclusions

The findings of the current study show that the methodology choices have an important impact on the estimated efficiency scores, implying that results of these studies should be interpreted and treated with caution. Moreover, the impact of modelling choices on efficiency scores in Iranian hospitals was comparable with that found internationally [6]. The studies included in this review suffer from major methodological deficiencies and are of sub-optimal quality, limiting their validity and reliability for policy-making in the country. Hollingworth [9] in his review of the efficiency studies in health care concluded that most studies in this field are of the “have software-will analyse” nature. Our review suggests the same scenario among Iranian studies. Including data on a broader range of hospital functions and quality of care in the Iranian hospital databases, and promoting the knowledge about hospital functions among researchers, as well as making better use of available data, and developing a critical assessment tool to evaluate the quality of efficiency studies are some major steps which should be taken to improve the quality of hospital efficiency studies in Iran and possibly other developing countries.

## Competing interests

The authors declare that they have no competing interests.

## Authors’ contributions

AAK participated in the design of the study, literature search, analysis and interpretation, and drafting of the manuscript. MJ participated in the literature search, interpretation and drafting of the manuscript. U-GG participated in the interpretation of data and drafting of the manuscript. All authors read and approved the final manuscript.

## Pre-publication history

The pre-publication history for this paper can be accessed here:

http://www.biomedcentral.com/1472-6963/13/312/prepub
